# Can the MRI based AMADEUS score accurately assess pre-surgery chondral defect severity according to the ICRS arthroscopic classification system?

**DOI:** 10.1186/s40634-022-00511-w

**Published:** 2022-08-19

**Authors:** Tizian Heinz, Felix Meller, Karsten Sebastian Luetkens, Konstantin Horas, Thomas Schäfer, Maximilian Rudert, Stephan Reppenhagen, Manuel Weißenberger

**Affiliations:** 1 Department of Orthopaedic Surgery, University of Wuerzburg, Koenig-Ludwig-Haus, Brettreichstr 11, 97074 Wuerzburg, Germany; 2 Department of Diagnostic and Interventional Radiology, University Hospital Wuerzburg, Oberduerrbacherstr 6, 97080 Wuerzburg, Germany

**Keywords:** Knee, Cartilage defect, Grading system of chondral defects, AMADEUS, ICRS, MRI

## Abstract

**Purpose:**

The AMADEUS (Area Measurement And DEpth and Underlying Structures) scoring and grading system has been proposed for the MRI based evaluation of untreated focal chondral defects around the knee. The clinical practicability, its correlation with arthroscopically assessed grading systems (ICRS – International Cartilage Repair Society) and thereby its clinical value in terms of decision making and guiding prognosis was yet to determine.

**Methods:**

From 2008 to 2019 a total of 89 individuals were indicated for high tibial valgus osteotomy (HTO) due to tibial varus deformity and concomitant chondral defects of the medial compartment of the knee. All patients received a preoperative MRI (1.5 Tesla or 3.0 Tesla) and pre-osteotomy diagnostic arthroscopy. Chondral defects of the medial compartment were scored and graded with the MRI based AMADEUS by three independent raters and compared to arthroscopic defect grading by the ICRS system. Interrater and intrarater reliability as well as correlation analysis with the ICRS classification system were assessed.

**Results:**

Intraclass correlation coefficients for the various subscores of the AMADEUS showed an overall good to excellent interrater agreement (min: 0.26, max: 0.80). Intrarater agreement turned out to be substantially inferior (min: 0.08, max: 0.53). Spearman correlation revealed an overall moderate correlative association of the AMADEUS subscores with the ICRS classification system, apart from the defect area subscore. Sensitivity of the AMADEUS to accurately identify defect severity according to the ICRS was 0.7 (0.69 for 3.0 Tesla MRI, 0.67 for 1.5 Tesla MRI). The mean AMADEUS grade was 2.60 ± 0.81 and the mean ICRS score 2.90 ± 0.63.

**Conclusions:**

Overall, the AMADEUS with all its subscores shows moderate correlation with the arthroscopic chondral grading system according to ICRS. This suggests that chondral defect grading by means of the MRI based AMADEUS is well capable of influencing and guiding treatment decisions. Interrater reliability shows overall good agreement.

## Background

With osteoarthritis being one of the most common degenerative joint disorders, especially affecting the knee, a variety of treatment strategies addressing the recovery of the joint function have evolved. Amid this plethora of treatment modalities, ranging from realignment techniques around the knee to total knee arthroplasty (TKA), practitioners are consistently challenged with selecting the most appropriate therapeutic pathway. One of the most essential parameters guiding treatment decision is the disease severity which includes a meticulously grading of cartilage wear and deterioration. In recent decades magnetic resonance imaging (MRI) with its high soft tissue contrast has evolved to the non-invasive imaging modality of choice for the evaluation of the chondral surface of the knee joint [4, 8, 6]. Since then, progress in MRI sequences has facilitated the evaluation of the knee joint cartilage by enhancing the visibility of early structural changes of the chondral surface. However, the sensitivity of the MRI to detect chondral lesions varies widely ranging from 0 to 100% depending on the location, size, depth of the lesions and the MRI sequence used [5, 6, 16, 21]. Another common problem besides the inconsistent sensitivity of the MRI resides in the lack of consistent and accurate classification systems of MRI diagnosed chondral lesions, hampering treatment decisions and total prognosis [13, 15]. While there are multiple MRI based classification systems for evaluation and assessment of knee osteoarthritis as well as for assessment of cartilage repair tissue, sound classification systems for the untreated, focal chondral lesion of the knee are lacking [12, 19, 20, 23]. Recently, Jungmann et al. reported on a novel MRI based chondral grading system for assessment of preoperative cartilage defect severity (AMADEUS – Area Measurement and DEpth and Underlying Structures) which is thought to guide treatment decision and prognosis of preoperatively encountered focal chondral lesions during routine knee MRI [15].

The aim of this study was to assess the clinical value of the proposed AMADEUS score and its correlation with the arthroscopic chondral defect assessment by means of the ICRS (International Cartilage Repair Society) grading system. Furthermore, the interrater reliability of the novel score was to evaluate.

It was hypothesized that the novel AMADEUS would be of great clinical value by demonstrating diagnostic accuracy, reliability and reproducibility.

## Methods

### Study population and design

This retrospective study encompassed a total of 89 patients at a single orthopaedic university center which were retrospectively analyzed by means of digitally archived medical records. All included individuals were indicated for surgery and received a high tibial valgus osteotomy (HTO) due to tibial varus deformity and ongoing pain of the medial knee compartment. Inclusion criteria were defined as follows: ongoing knee pain predominantly of the medial compartment, varus deformity of the tibial plateau and a preserved and intact lateral knee compartment. For eligibility of this retrospective study, patients were required to have a latest MRI (1.5 Tesla or 3.0 Tesla) of their knee before surgery as well as a diagnostic arthroscopy of the concerned knee joint. In addition, the presence of a cartilage defect of the medial compartment either diagnosed arthroscopically or by MRI was stringent. From November 2008 through September 2019 a total of 89 patients were found to be eligible and were retrospectively evaluated. The study was approved by the local institutional review board. The requirement for informed consent was waived.

### MR Image Analysis und Assessment

**Fig. 1:**
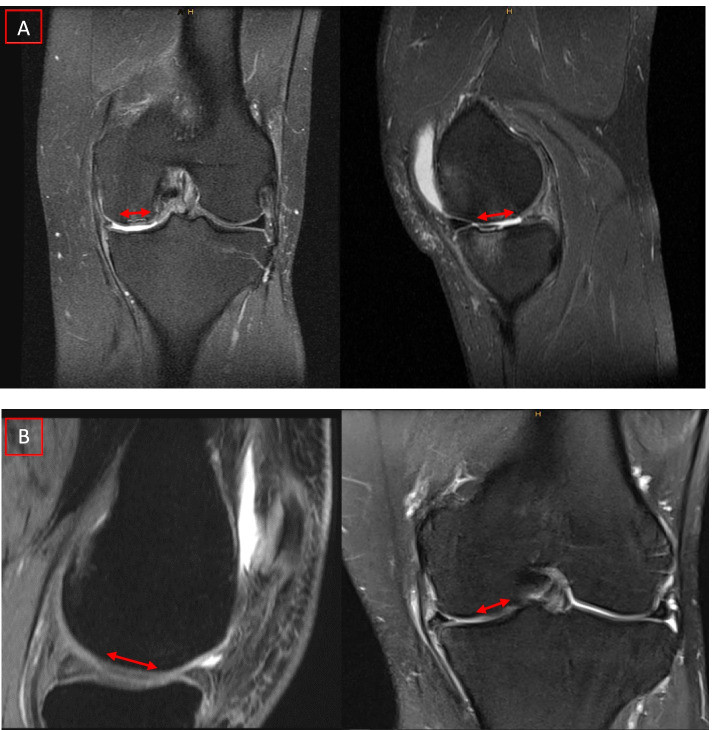
Examples of cartilage defect assessment on MRI and grading according to the AMADEUS instruction guide. **A**) Example of a full thickness cartilage defect with an associated bone marrow edema and small (< 5 mm bony defect) scoring for an AMADEUS grade IV. **B**) Example of a superficial cartilage defect with intact subchondral bone and absent bone marrow edema scoring for an AMADEUS grade II

All MRI images were digitally stored and accessible via Picture Archiving Communication System (PACS). Inclusion criteria encompassed a preoperatively obtained 1.5 T (21 patients) or 3.0 T MRI (68 patients) of the knee joint in at least two planes. MR images had to be either of an intermediate-weighted (IM) or T2-weighted fast spin echo (FSE) sequence or a proton-density (PD) weighted sequence in combination with an T2-weighted FSE in at least two planes, respectively. Furthermore, for the evaluation of the bone marrow a T1-weighted images was additionally required. MR sequences with a higher spatial resolution like the spoiled gradient echo (SPGR) sequence have also been found eligible if available in at least two planes. All MR images were independently evaluated by three different raters: One radiologist trained in musculoskeletal imaging, one resident trained in orthopaedics and traumatology and another intern in orthopaedics. Furthermore, for evaluating the intraobserver reliability, one rater was chosen to evaluate the MRI images six months after the initial rating. Cartilage defects of the medial compartment were evaluated on the MR images according to the instructional of the AMADEUS score for the assessment of preoperative cartilage defect severity [15]. Defect size, defect depth, integrity of the underlying structure and the presence of a concomitant bone marrow edema were evaluated for every single cartilage defect (Fig. 1). One rater additionally assessed the Kellgren-Lawrence-Score (KLS) of the medial compartment on plain preoperatively obtained radiographs of the knee. However, this rater assessed the AMADEUS and Kellgren-Lawrence-Score independently in a random order of the patients.

### Statistical analysis

Statistical analysis was performed using SPSS statistical software (SPSS, Chicago, IL, USA, Version 27). A p-value of 0.05 was set as level of significance. Variables were analyzed using descriptive statistics including mean values with standard deviations and frequencies. Data was checked for normal distribution using Kolmogorov–Smirnov and Shapiro–Wilk test. If data were lacking normal distribution non-parametric testing was performed. For interrater reliability of the AMADEUS the intraclass correlation coefficients (ICC) were calculated for all three raters and for all AMADEUS subscores respectively. ICC was determined using SPSS statistical package version 27 (SPSS, Chicago, IL, USA) based on a mean-rating (k = 3), consistency, two-way random-effects model. An ICC of less than 0.5, 0.5 to 0.75, 0.75 to 0.9 and above 0.9 were interpreted as poor, moderate, good and excellent reliability based on current literature of Koo et al. [17]. For intrarater reliability the ICC was calculated based on an absolute agreement, two-way mixed-effects model. A Spearman correlation analysis was performed to evaluate strength and association between the MR based AMADEUS score and the arthroscopic based ICRS score. The strength of the Spearman correlation coefficients were interpreted based on the findings of Chan et al. with values between 0.1 and 0.3 indicating a weak, between 0.4 and 0.6 a moderate, between 0.7 and 0.9 a strong and values above 0.9 a perfect correlation [2]. A Fisher Z-transformation was then performed to compare the correlation coefficients for differences in the distribution patterns of the AMADEUS grade assignments between the three raters.

Sensitivity of the AMADEUS was determined by comparing the ratings to the ICRS grade. True positive cases were assumed if the AMADEUS grade and the ICRS grade of a subject were identical. False negative cases were defined for subjects with a lower (i.e. better) AMADEUS grade compared to the corresponding ICRS grade. A sub-analysis for patients with 1.5 T and 3.0 Tesla MRI was made and tested for statistical significance using the Chi-Square Test.

## Results

**Fig. 2:**
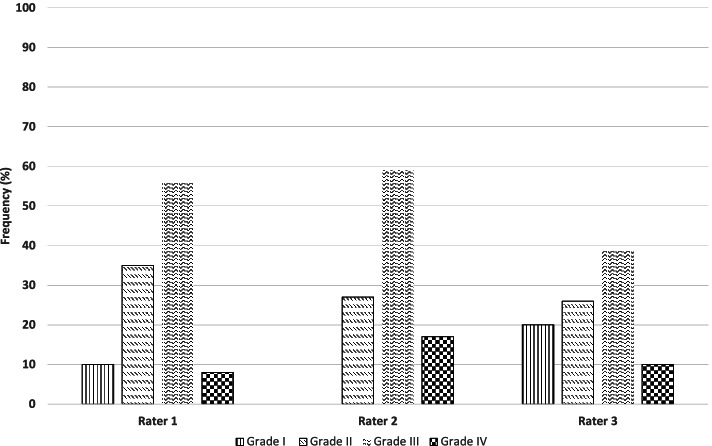
The distribution of the AMADEUS grade assignments among rater 1 to rater 3

A total of 89 patients were retrospectively evaluated with a mean age of 44.06 ± 8.72 years. There was a predominance of male patients (93.3% vs. 6.7%). The distribution of the Kellgren-Lawrence, ICRS and AMADEUS grades are depicted in Table 1. Mean Kellgren-Lawrence grade turned out to be 1.37 ± 0.55, compared to the mean AMADEUS grade (2.60 ± 0.81) and ICRS grade (2.90 ± 0.63). The mean defect area measurement on MRI was 2.49 ± 1.9 cm^2^. The intraclass correlation coefficients (ICC) for interobserver agreement are depicted in Table 2. The highest ICC was found for the agreement on existing bone marrow edema (BME) around the cartilage defect (ICC = 0.80) indicating a very good interobserver reliability, followed by the second highest ICC for the AMADEUS total score (ICC = 0.71). The ICC for intraobserver agreement are depicted in Table 3. The distribution of the AMADEUS grade assignments among rater 1, rater 2 and rater 3 are shown in Fig. 2. Chi-square analysis revealed no statistically significant differences in the distribution patterns among rater 1 to rater 3 regarding the AMADEUS grade assignments. Furthermore, a Chi-square analysis for independency was performed for the subscores of the AMADEUS with the ICRS score, revealing a dependency only for the AMADEUS grade (X^2^ (9) = 18.94, *p* = 0.03), AMADEUS total score (X^2^ (27) = 0.04), AMADEUS depth score (X^2^ (6) = 17,47, *p* = 0.01) and AMADEUS underlying structure score (X^2^ (6) = 14.99, *p* = 0.02). However, the AMADEUS defect area score did not show any association with the ICRS score. A correlation analysis of dependent variables of the AMADEUS score with the ICRS score was performed as shown in Table 4. Apart from the defect area score and BME variable, all AMADEUS variables showed a moderate correlation with the ICRS score. Correlation coefficients of the AMADEUS total score with the ICRS score did not differ significantly among the raters (z = 0.32 – 1.19, *p* = 0.12 – 0.37). The same applied to the correlation coefficients of the AMADEUS grade with the ICRS score among the raters (z = -0.29—-0.99, *p* = 0.18 – 0.39). The highest correlation was observed for the AMADEUS depth subscore. However, correlation analysis of the KLS grade and the ICRS score did not reveal a significant association (r = 0.14 p = 0.20). Furthermore, the KLS grade did not show any correlation with the AMADEUS grade. Table 5 demonstrates the mean values of the AMADEUS subscores for each rater respectively. Significant differences in the mean values within a specific AMADEUS subscore, as scored differently by the raters, were calculated (Table 5). Overall sensitivity of the AMADEUS to accurately predict defect severity according to the ICRS classification turned out to be 0.70. In case of a 3.0 T MRI performed before surgery, the sensitivity turned out to be 0.69, compared to 0.67 for the 1.5 T MRI. However, this difference turned out not to be of statistical relevance (X^2^ = 0.78, *p* = 0.85, df = 3, *n* = 206).

**Table 1:** Descriptive statistics of the study cohort

	**Mean (± SD) or frequency (%)**
Age	44.06 ± 8.72
Sex	Total *n* = 89 (100%)
Male	93.3%
Female	6.7%
Side of surgery	
Right knee	43.8%
Left knee	56.2%
Kellgren-Lawrence grade distribution	
Grade I	66.3%
Grade II	30.3%
Grade III	3.4%
Grade IV	0%
AMADEUS grade distribution	
Grade I	10.2%
Grade II	29 7%
Grade III	50.0%
Grade IV	10.2%
ICRS grade distribution	
Grade I (Ia / Ib)	3.4%
Grade II	14.6%
Grade III (IIIa – IIId)	68.5%
Grade IV (IVa – IVb)	11.5%

**Table 2:** Intraclass correlation coefficients for interobserver agreement

	**Interobserver Agreement (95% CI) (ICC)**
AMADEUS grade	0.70 (0.30 – 0.56)
AMADEUS total score	0.71 (0.59 – 0.80)
Score “defect area”	0.54 (0.35 – 0.69)
Score “depth”	0.60 (0.43 – 0.73)
Score “underlying structure”	0.26 (-0.08 – 0.50)
Score “BME”	0.80 (0.71 – 0.86)

**Table 3:** Intraclass correlation coefficients for intraobserver agreement

	**Intraobserver Agreement (95% CI) (ICC)**
AMADEUS grade	0.33 (- 0.02 – 0.56)
AMADEUS total score	0.42 (0.11 – 0.62)
Score “defect area”	- 0.08 (- 0.64 – 0.29)
Score “depth”	0.39 (0.06 – 0.60)
Score “underlying structure”	0.53 (0.28– 0.69)
Score “BME”	0.49 (0.22 – 0.66)

**Table 4:** Spearman correlation analysis of dependent AMADEUS subscore items with ICRS classification

AMADEUS variables	Correlation	ICRS
AMADEUS total score	Coefficient	- 0.32
	Significance	**0.00**
	Number	89
AMADEUS “defect area”	Coefficient	- 0.21
	Significance	0.06
	Number	89
AMADEUS “depth”	Coefficient	- 0.36
	Significance	**0.00**
	Number	89
AMDEUS “underlying structure”	Coefficient	- 0.23
	Significance	**0.04**
	Number	89
AMADEUS “BME”	Coefficient	- 0.20
	Significance	0.06
	Number	89
AMADEUS grade	Coefficient	0.31
	Significance	**0.00**
	Number	89

**Table 5:** Mean values of the AMADEUS subscores for each rater respectively. A Kruskal–Wallis-Test was performed to reveal significant differences in the mean values within an AMADEUS subscore for different raters

	**Rater 1 (mean ± SD)**	**Rater 2 (mean ± SD)**	**Rater 3 (mean ± SD)**	**Mean-Value (rater 1 to rater 3)**	***p*-Value Rater 1 – Rater 3**	***p*-Value Rater 1 – Rater 2**	***p*-Value Rater 2 – Rater 3**
**AMADEUS total score**	52.64 ± 17.5	41.93 ± 13.31	56.01 ± 21.67	50.11 ± 14.23	0.78	**0.00**	**0.00**
**Score “defect area”**	18.09 ± 8.77	31.70 ± 4.14	24.16 ± 9.60	24.64 ± 5.69	**0.00**	**0.00**	**0.00**
**Score “depth”**	5.11 ± 5.23	9.32 ± 5.98	5.11 ± 6.17	6.46 ± 4.32	0.97	**0.00**	**0.00**
**Score “underlying structure”**	26.63 ± 8.39	26.59 ± 8.43	26.25 ± 4.87	27.09 ± 4.04	**0.00**	**0.00**	0.97
**Score “BME”**	2.81 ± 4.52	5.00 ± 5.03	3.15 ± 4.67	3.67 ± 4.01	0.64	**0.01**	**0.03**

## Discussion

Over the last decades, MRI has evolved to the radiographic imaging modality of choice for detection of intraarticular pathologies of the knee joint [9, 10, 26]. While the diagnostic accuracy for detection of meniscal and anterior cruciate ligament disorders is generally considered to be high, profound evaluation of the hyaline cartilage of the knee joint by MRI remains challenging [3, 22, 28]. Aside from the demanding task to enable the visibility of chondral lesions by adequate MRI sequences and protocols, another common difficulty is based on the lack of sound classification systems for proper radiographic chondral grading. While a variety of radiographic classification systems involve the evaluation of cartilage defects, most of them have not been designed to assess focal chondral defects prior to surgery. For instance, the widely applied MOCART (Magnetic Resonance Observation of Cartilage Repair Tissue) score is specifically designed to evaluate the cartilage repair tissue following cartilage repair surgery [18, 25]. The Whole Organ Magnetic Resonance Imaging Score (WORMS), MRI Osteoarthritis Knee Score (MOAKS), Boston Leeds Osteoarthritis Knee Score (BLOKS) and Cartilage Lesion Score (CaLS) are radiographic grading systems that aim for a holistic evaluation of the whole osteoarthritic knee joint rather than focusing on a single focal cartilage defect [1, 11, 12, 23, 27]. Meanwhile, for the description and evaluation of pre-surgery chondral defects macroscopic classification systems like the ICRS grading system have been transferred to MR image evaluation, though never been developed specifically for this imaging modality [14]. Therefore, the novel AMADEUS is thought to fill this gap of lacking radiographic grading systems for pre-surgery chondral defects. By implementing the subscores “cartilage defect size”, “defect depth” and “subchondral bone involvement”, the AMADEUS is thought to accurately portray defect severity and to aid in clinical decision-making regarding cartilage repair surgery. The AMADEUS score has already been linked to clinical data demonstrating moderate correlation with patient reported outcomes measures (PROMs) [24]. However, its association with arthroscopic chondral defect grading systems has not been investigated yet.

In the present study it could be demonstrated that the AMADEUS total score as well as the AMADEUS grading scale provide a moderate correlation with the arthroscopic based ICRS classification system. This finding is of high relevance, as to date the arthroscopic evaluation of chondral defects is still considered the method of choice in terms of diagnostic accuracy [7, 28]. Therefore, clinical decision-making and cartilage repair guidelines mainly rely on macroscopic classification systems like Outerbridge and ICRS (International Cartilage Repair Society). However, the correlation of the AMADEUS score with the ICRS classification suggests that radiographic grading of chondral defects is well capable of influencing and guiding treatment decisions. This is also in line with the high sensitivity of the AMADEUS to accurately predict cartilage defect severity according to the ICRS grade. By being comparable to the ICRS classification, practitioners can easily imagine and predict the defect severity of a chondral lesion without the stringent requirement of an additional diagnostic arthroscopy. However, due to the varying sensitivity of the MRI for detection of chondral defects, a diagnostic arthroscopy still seems indicating in some cases, especially if high clinically suspicion with a simultaneously negative MRI rating exists [7]. Evaluation of the AMADEUS further facilitates interdisciplinary communication and allows for multicenter comparisons in patient registries by making radiologically assessed chondral defects more comparable [15]. Surprisingly, the defect area score as part of a subscore of the total AMADEUS score did not show any correlation with the ICRS classification. This finding may seem incongruous at first glance, but as the ICRS classification does not take the defect size as a separate parameter into account, a correlative association is unlikely to be expected. Apart from the defect area subscore, all remaining subscores of the AMADEUS total score showed a moderate to strong correlative association with the ICRS classification, easing comparability and therapeutic guidance. Jungmann et al. reported on a high interobserver reliability of the AMADEUS score with all its subscores [15]. However, comparable studies verifying the observed interclass correlations are sparse. Therefore, in the present study three raters were randomly selected to evaluate the chondral defects on MRI according to the AMADEUS scoring guideline. All three raters were at different stages of their orthopaedic training, one intern in orthopaedics, another resident in orthopaedic surgery and a fully trained radiologist specialized in musculoskeletal imaging. Raters of different skill levels were chosen by purpose to evaluate whether the skill and training level may bias the final AMADEUS scoring. Interestingly, correlation coefficients did not differ significantly between the raters suggesting that the AMADUES score is resistant to different training levels of the readers.

Interestingly, in this study intraobserver agreement turned out to be substantially inferior to the interobserver reliability. This is in contrast to the study by Jungman et al. reporting on a high overall intraobserver agreement [15]. For this reason, further research focusing on intrarater reproducibility of the AMADEUS seems necessary.

Furthermore, this retrospective analysis did not reveal any correlative association of the KLS with the AMADEUS grade or ICRS grade. This is fairly expectable, as the AMADEUS and ICRS intend to assess focal cartilage defects of the knee joint which can be generally considered as a prearthrotic joint disease, though having the potential of evolving into a degenerative joint destroying process later on.

However, with this study there are also some shortcomings that need to be considered. As all included individuals underwent HTO as the one and only operative procedure, chondral defect patterns are likely to be similar among the study population. Diversifying the study population in terms of the applied surgical procedure would have diminished a potential bias. However, compared to similar studies regarding this topic, the number of included individuals is remarkedly higher, adding strength and value to this research [15, 24]. Furthermore, there was a mixed patient cohort in terms of the Tesla strength (1.5 and 3.0 Tesla) of the pre-surgery MRI. This circumstance may add further bias, but still reflects a typical clinical scenario found in daily routine.

## Conclusions

Overall, the AMADEUS with all its subscores shows moderate correlation with the arthroscopic chondral grading system according to ICRS. This suggests that chondral defect grading by means of the MRI based AMADEUS is well capable of influencing and guiding treatment decisions. Interrater reliability shows overall good agreement.

## Data Availability

The datasets used and/or analysed during the current study are available from the corresponding author on reasonable request.

## Abbreviations

AMADEUS: Area Measurement And DEpth and Underlying Structure
KLS: Kellgren-Lawrence-Score
MOCART: Magnetic Resonance Observation of Cartilage Repair Tissue
ICRS: International Cartilage Repair Society
